# Risk factors related to oral candidiasis in patients with primary Sjögren’s syndrome

**DOI:** 10.4317/medoral.23719

**Published:** 2020-07-19

**Authors:** Julia Serrano, Rosa María López-Pintor, Lucía Ramírez, Mónica Fernández-Castro, Mariano Sanz, Sheila Melchor, Diana Peiteado, Gonzalo Hernández

**Affiliations:** 1 Department of Dental Clinical Specialties, ORALMED research group, School of Dentistry, Complutense University, Madrid, Spain; 2 PhD Student, Research training fellow, Complutense University, Madrid; 3 Rheumatology Service, Hospital Puerta de Hierro, Madrid, Spain; 4 Rheumatology Service, Hospital Doce de Octubre, Madrid, Spain; 5 Rheumatology Service, Hospital La Paz, Madrid, Spain

## Abstract

**Background:**

Candidiasis is the most frequent mycotic infection of the oral cavity. The aim of this study was to investigate the presence of clinical oral candidiasis and Candida albicans yeast in a population diagnosed of primary Sjögren’s syndrome (pSS) and to study the possible factors associated with this infection.

**Material and Methods:**

An observational cross-sectional study was conducted in 61 pSS patients (60 women, 1 man, mean age 57.64±13.52) where patient based information (demographic and medical, tobacco and alcohol consumption history), intraoral parameters (presence of dentures, clinical signs of candidiasis), salivary analytical information (number of *Candida albicans* as colony-forming units per millilitre (CFU/mL), salivary pH levels, unstimulated whole saliva (UWS) and stimulated whole saliva (SWS) were collected.

**Results:**

13.1% of pSS patients presented oral signs of candidiasis. Denture stomatitis and angular cheilitis were the most common lesions. 87.5% of patients with clinical candidiasis presented reduced pH levels and salivary flow in both UWS and SWS. A significant statistical negative correlation was found between CFU/mL of *Candida albicans* and levels of UWS and SWS. A negative correlation was found between pH levels and CFU/mL, although not statistically significant.

**Conclusions:**

A reduced salivary flow may predispose pSS patients to *Candida albicans* overgrowth, which may show with clinical signs. Preventive measures are of great importance to avoid and to treat this condition promptly.

** Key words:**Sjögren’s syndrome, oral candidiasis, oral lesions, Candida albicans, oral yeast, salivary flow rate, hyposalivation.

## Introduction

Primary Sjögren’s syndrome (pSS) is a systemic autoimmune exocrinopathy that damages the salivary and lacrimal glands, resulting in dry eyes and hyposalivation (1,2). Saliva contains IgA, lysozyme and lactoferrin, which are important antimicrobial defence mechanisms. Moreover, proper levels of saliva allow the lubrication of the mucosa and its buffering capacity maintains a physiological pH within the oral cavity (3,4). In pSS patient’s salivary gland hypofunction reduces the concentrations of immunoglobulins and other electrolytes (5), thus making the mucous membranes more exposed to the oral microbiota, and specifically to *Candida* infections (6). *Candida* species are commensal yeast present in the oral flora of healthy population. Nevertheless, in SS patients its prevalence has been estimated to be higher (7,8). Therefore, simple identification of *Candida* yeast does not prove any infection and it is not always associated with the presence of clinical oral candidiasis (9). Candidiasis is the most frequent mycotic infection of the oral cavity, and it is usually diagnosed clinically, based on recognition of related lesions (9). The pathogenesis of this infection is still not fully understood, but a variety of systemic (as immunosuppression or endocrine disorders) and local factors (reduced salivary flow, use of dentures, high sugar diet, among others) have been associated to an overgrowth of *Candida* species, being *Candida albicans* the species most often associated with oral lesions (10,11). This variety of predisposing factors alters to an environment that favours proliferation of *Candida* and leads to its transition from commensal to pathogenic, which may show with clinical signs and symptoms of oral candidiasis (9). The reported prevalence of clinical oral candidiasis in SS has varied widely (0%-80%), mainly due to three factors: the lack of a clear symptomatology, patient related factors (such as oral hygiene habits) and different criteria used for diagnosing oral candidiasis in the literature (12).

Therefore, the main objective of this observational study was to investigate in a cohort of patients with pSS the association between the presence of *C. albicans* and clinical lesions of oral candidiasis with their salivary flow rates and pH levels. We also studied the possible influence of patient-related factors in the development of clinical oral candidiasis.

## Material and Methods

- Study design, setting and subjects

A cross-sectional observational study was conducted following STROBE guidelines, as part of the EPOX-SSp project (13). The patient cohort was the same as in a previous study carried out by this research group (14). This sample consisted on consecutive patients who attended at different rheumatology services in the Community of Madrid (Spain) and which met the following inclusion criteria: being over 18 years old and being diagnosed of pSS according to the diagnostic criteria proposed by the American European Consensus Group (AECG) in 2002 (15). If selected patients had any other connective tissue disease or difficulties to attend to the School of Dentistry were excluded.

- Clinical variables and clinical diagnosis of oral candidiasis

A standard clinical protocol was applied and the following variables were recorded:

a) Patient related: age and gender, medical history, type and number of medicines, alcohol and tobacco consumption and wear and type of denture.

b) pSS disease: pSS AECG-2002 diagnostic criteria, time from diagnosis, serological data (rheumatoid factor, immunoglobulins alteration, antinuclear autoantibodies), and systemic manifestations of pSS (parotid enlargement, musculoskeletal, skin, lung, renal, central nervous system, peripheral nervous system, haematological, gastrointestinal or cardiac involvement).

c) Oral symptoms: xerostomia, dysphagia, dysgeusia and glossodynia.

A complete facial and intraoral mucosa examination was carried out by one calibrated specialist in oral medicine (JS). Diagnosis of clinical oral candidiasis was based on presence of the following clinical presentations: pseudomembranous candidiasis, acute erythematous candidiasis, chronic erythematous candidiasis (denture stomatitis), chronic hyperplastic candidiasis and secondary forms of oral candidiasis (angular cheilitis, median rhomboid glossitis and chronic mucocutaneous candidiasis) (11).

- Saliva Sampling

Stimulated (SWS) and unstimulated saliva (UWS) were collected by one specialist in oral medicine (LR). Patients were told not to eat, drink, smoke or brush their teeth 90 minutes prior the appointment. Samples were obtained in the morning, between 8.00 and 10.00 am, with the UWS sample always collected first during 15 minutes. To collect the SWS sample patients were asked to chew a paraffin gum and continuously spit out the saliva into a plastic container for 10 minutes. Flow rates were recorded as mL/min. Hyposalivation was defined as a flow rate <0.7 mL/min for SWS and <0.1 mL/min for UWS (14). To measure the salivary pH a pH meter was used (MicroPh2001, CRISON).

- Microbial sampling, culturing, and quantification of yeasts

Microbial samples were taken by rubbing a sterile cotton swab along the dorsal surface of the tongue of all patients. All swabs were taken by one operator (JS) before the saliva collection. Samples were transferred into a vial containing 1mL RTF (reduced transport fluid), vortexed for 30 seconds in a mixer for homogenizing the collection and then aliquots of 0.02mL of suspension were plated onto Sabouroud Agar plates (Sabouraud Dextrose Agar- DIFCO) and incubated at 37°C for 24-48 hours. After incubation, colony-forming units per millilitre (CFU/mL) of *C. albicans* were counted. All samples were analysed at the Microbiology Laboratory of Faculty of Odontology at the University Complutense of Madrid.

- Statistical analysis

The sample size calculation (shown in a previous study by the same research group) (14) was calculated considering the data on prevalence of oral candidiasis (29.9%) reported by Likar-Manookin *et al*. (3). Since this study did not compare oral candidiasis with a control group we used the percentage of oral candidiasis of the study of López-Pintor *et al*., which also compared the presence of oral candidiasis in renal transplant patients with a control group (4.19%) using the same methodology (16). If we consider an α =0.05 and a statistical power of 90%, 35 patients will be needed in each group. If we consider a 15% loss, 41 patients will be needed per group.

The categorical variables were described by their number and percentage and the quantitative variables by their media ± standard deviation (SD). The Kolmogorov-Smirnoff test was applied to establish their goodness of fit to normality. To determine the differences between those pSS patients suffering from clinical oral candidiasis from those not suffering in regards to the categorical variables, the chi-squared test or Fisher’s exact test were used. When assessing the differences in the quantitative variables, the U Mann-Whitney test was used. Spearman’s rank correlation (r) coefficients were calculated to explore the associations between the quantitative variables. Differences were considered significant if *p* was ≤ 0.05. All data were analysed using SPSS 25.0 for Windows.

## Results

- Participants and descriptive data

All demographic data, pSS characteristics, systemic and serological manifestations from the selected patients are depicted in Table 1.

- Oral candidiasis in pSS

Clinical signs of oral candidiasis were present in 13.1% of pSS patients. Denture stomatitis was the most prevalent lesion (8.2%), followed by angular cheilitis (4.9%) and median rhomboid glossitis (1.6%). No relationship was found between the clinical signs of oral candidiasis and the pSS variables. No other associations were observed between the rest of variables and the presence of clinical oral candidiasis (Table 1).

- Oral candidiasis and salivary flow rates

Table 2 shows the association between the presence of clinical oral candidiasis and UWS and SWS flow rates as well as with hyposalivation. pSS patients with oral candidiasis suffered more UWS and SWS hyposalivation and had lower flow rates compared with those without clinical oral candidiasis. A statistically significant association between clinical oral candidiasis and SWS hyposalivation was found.

- CFU/mL, salivary flow rates and pH levels

**Figure 1:**
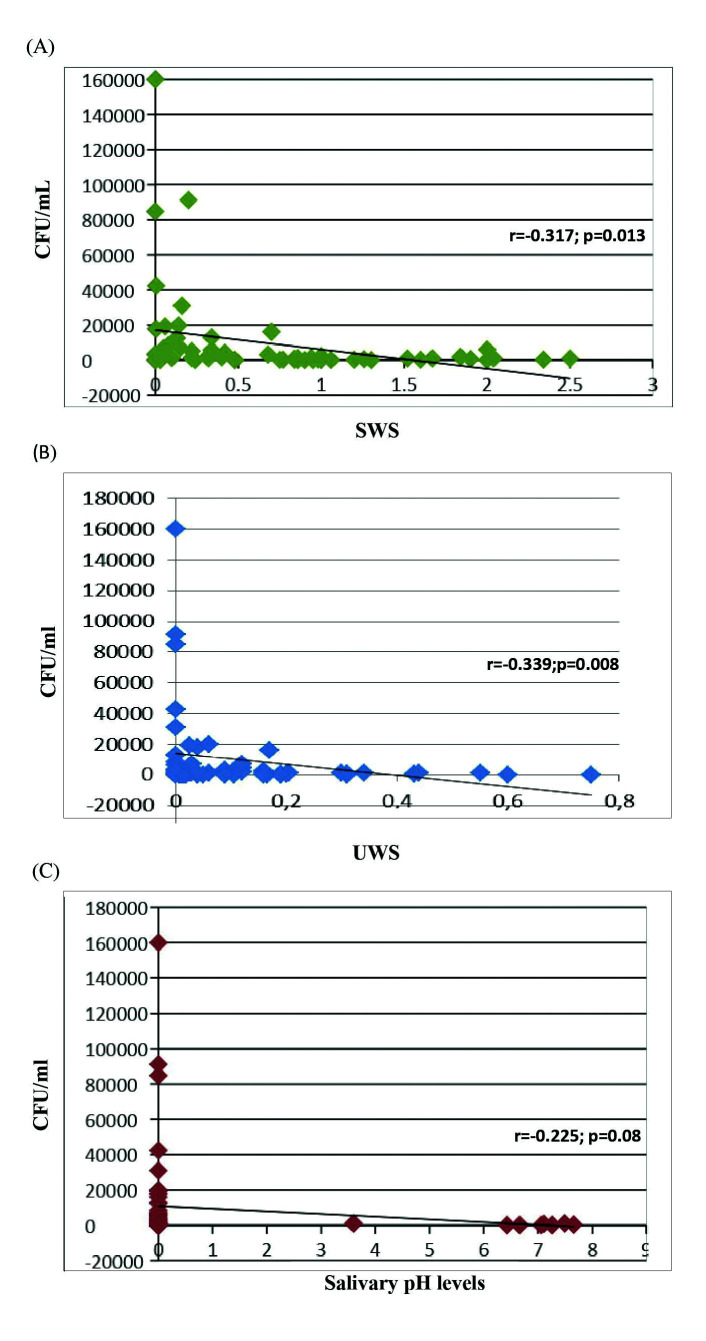
(A) Correlation between SWS and *Candida albicans* CFU/mL, (B) Correlation between UWS and *Candida albicans* CFU/mL, (C) Correlation between salivary pH levels and *Candida*.

A statistically significant negative correlation was found between the amounts of *C. albicans* in CFU/mL and the salivary flow levels of SWS (r=-0.317; *p*=0.013) and UWS (r=-0.339; *p*=0.008) (Fig. 1). A negative correlation between *C. albicans* and salivary pH levels was also found (r=-0.225; *p*=0.08), but without reaching statistical significance (Fig. 1).

**Table 1:**
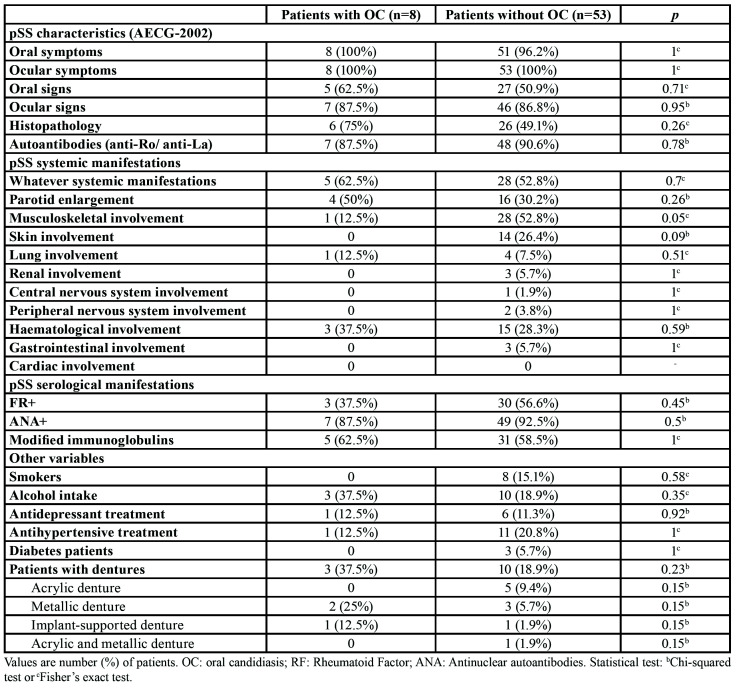
Differences between patients with and without oral candidiasis (OC).

**Table 2:**
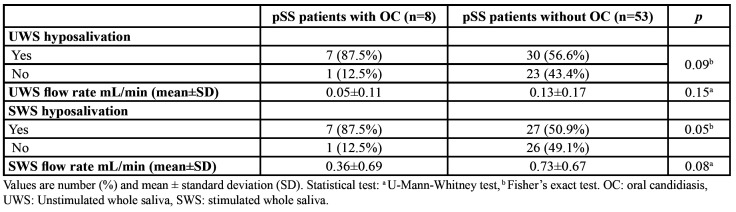
Relationship between oral candidiasis and salivary variables.

- Oral candidiasis and oral symptoms

100% of pSS patients with clinical oral candidiasis also suffered xerostomia (*p*=0.3), 50% dysphagia (*p*=0.7) and glossodynia (*p*=0.27), but none referred dysgeusia (*p*=0.28). The association between having clinical oral candidiasis and symptoms of oral discomfort was not statistically significant, although xerostomia was always present.

## Discussion

This cross-sectional study on a cohort of pSS patients has found a significant inverse relationship between UWS and SWS levels and *C. albicans* counts. Hyposalivation is one of the main manifestations of SS. When salivary secretion is diminished, not only the volume of saliva decreases, but also its composition is altered, which may result in changes of the normal microflora homeostasis (17,18). Hence, the predisposition of patients with decreased salivary flow to oral candidiasis could be attributed not only to the lack of mechanical washing activity but also to the lack of protective salivary factors (19-21).

Hyposalivation has been associated with an increase in oral *Candida* counts (6), but, specifically in SS, it is not clear whether these high *Candida* levels are associated to lower UWS and/or decreased SWS flow rates. In fact, some authors have studied both types of saliva in pSS patients and have reported high *Candida* counts associated to both lower rates of SWS and UWS, although without demonstrating statistical significance (22,23). Similarly, there are studies reporting that when *C. albicans* is higher UWS is decreased (2,24,25), some demonstrating a significant relationship (2,25). In other studies, however, the association was with SWS rather than UWS (26). In the present study, we have analysed both UWS and SWS and we have found a significant inverse relationship with *C. albicans*. These previous and present results emphasize the importance to frequently monitor the salivary flow rates in pSS patients to prevent superinfections by *C. albicans*. The clinical results from this study have further confirmed that hyposalivation is associated with clinical oral candidiasis. 87.5% of patients with clinical oral candidiasis presented SWS hyposalivation, compared to 50.9% pSS patients with a healthy mucosa. Similarly, 87.5% of patients with oral clinical candidiasis had UWS hyposalivation, compared to 56.6% without. As in previous reports (25) we have observed that pSS patients with low salivary flow rates had a higher risk for developing clinical oral candidiasis, with a statistical association between the SWS flow rate and oral candidiasis, however not with the UWS. In regard to other variables studied, we did not find any association between pSS related outcomes and clinical oral candidiasis. This is in agreement with Billings *et al*. who found that hyposalivation both UWS and SWS was significantly associated with clinical oral candidiasis, but not with other variables such as presence of autoantibodies and focus score (7).

It is accepted that *Candida* adherence to epithelial and acrylic surfaces is enhanced in the presence of low pH levels and salivary flow rate (27), nevertheless, very few studies have reported values of pH levels in SS patients (5). To our knowledge, this is the first report that compares pH levels in pSS patients with clinical signs of candidiasis or *C. albicans* loads. Low salivary pH values in SS may be caused by the low salivary flow rates and the decreased buffering capacity, although it is also possible that the progressive destruction of the salivary gland tissue may have a direct effect on the pH salivary secretion. In this investigation pSS patients with clinical oral candidiasis had lower pH levels (4.40±2.91) than those without (5.19±2.80) although these differences were not statistically significant. Similarly, when studying the association between *C. albicans* counts and pH levels, the lower the salivary pH the higher were the *C. albicans* counts. The lack of a significant association may be due to the variable ranges in pH described for a favourable *Candida* growth, between 3 to 8 (28). Another explanation may be due to the confounding effect of oral hygiene products, such as toothpastes or mouthwashes that compensate the drop in pH in these patients. More articles are needed to elucidate the possible relationship between pH levels and buffer capacity with *Candida* overgrowth and infection.

Although high *Candida* counts are predictive for clinical oral candidiasis (6), there is no relevant information on the critical yeast counts necessary to evidence the clinical signs of oral candidiasis (29). A recent study published by Zhou *et al*. (29) concluded that 266 CFU/mL of *C. albicans* in saliva samples could be used as a threshold. In our study all patients with clinical signs of candidiasis had more than 266 CFU/mL. New studies where the relationship of *C. albicans* counts and clinical signs of oral candidiasis should be carried out, in order to unify a cut-off point for considering *C. albicans* counts as an infection.

The present study has some limitations. The first is the cross-sectional nature of this investigation. Another limitation is the lack of a control non-pSS group. Besides, it should be noted that the low rate of clinical oral candidiasis observed in the current study may be due to good oral hygiene of the patients studied, 90% of included pSS patients brushed their teeth at least twice a day.

In conclusion, this study shows how a lower salivary flow is related to higher counts of *C. albicans*. Since this oral condition may be symptomless, it is highly recommended the frequent monitoring and the emphasis of increased oral hygiene practices in these patients. Intervention prospective studies are needed to assess the impact of these enhanced oral preventive measures on the incidence of oral clinical candidiasis.
